# Q&A with Editorial Board Member Professor Kristin Wustholz

**DOI:** 10.1038/s42004-022-00776-3

**Published:** 2022-11-22

## Abstract

Professor Kristin Wustholz answers questions on her scientific career, scientific developments she is excited about and directions the spectroscopy and photochemistry communities should focus on, as well as her experience of being an Editorial Board Member for *Communications Chemistry*.

Kristin Wustholz obtained a B.A.S. in Chemistry and Philosophy from Muhlenberg College (USA) in 2002. She obtained her M.S. (2005) and Ph.D. (2007) from the University of Washington in Seattle, funded in part by an NSF IGERT fellowship. Her research with Bart Kahr and Phil Reid involved single-molecule spectroscopy of dyed salt crystals. As a postdoctoral fellow at Northwestern University, she studied plasmonics, localized surface plasmon resonance microscopy, surface-enhanced Raman spectroscopy (SERS), and single-molecule SERS with Richard Van Duyne.

**Figure:**
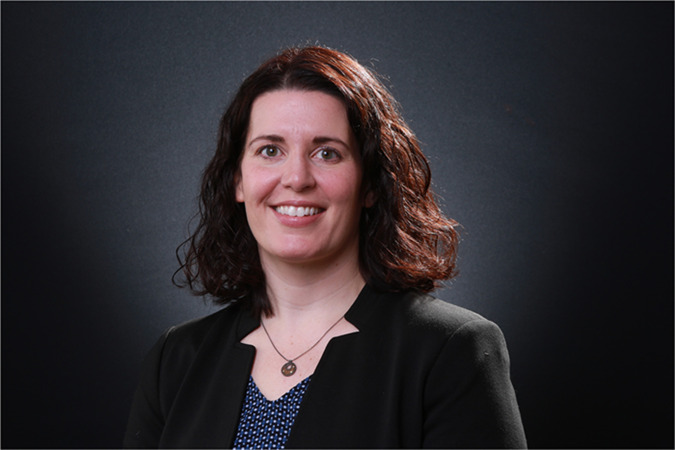
William & Mary

Kristin began her independent career at William & Mary (USA) in 2010, where she is currently the Mansfield Associate Professor of Chemistry. Her group uses single-molecule and surface-enhanced spectroscopies to probe the optical and structural properties of chromophores in environments that are inherently complex for applications to solar energy conversion and art conservation. Recently, her group discovered blinking-based multiplexing (BBM), a simple, versatile approach to multicolor super-resolved imaging without using spectrally distinct probes.

Why did you choose to be a scientist?

I have always been fascinated by science, even as a child. But I didn’t see myself as a scientist until I met my high school chemistry teacher, Carol Guogas. Carol was an utterly fantastic teacher – challenging, fair, kind, and motivating. She helped me see that my curiosity, academic accomplishments, and lab skills could readily translate into a scientific career. I distinctly remember the day we did flame tests in lab. The moment I saw rocks glow in vibrant hues, I knew that I wanted to be scientist studying light and color.

Fast forward to my time at Muhlenberg College, a small liberal arts institution, where I had the opportunity of working closely with Profs. Marsha Baar and Bruce Anderson in the research lab setting. From these tremendous mentors I experienced both the thrills and the frustrations of research in organic synthesis and photochemistry, finding ultimately, a deep joy in the process of figuring something out, sharing that knowledge with colleagues, and advancing the field. Now, as a faculty member at William & Mary, I have the distinct privilege of working with bright, diverse, curious, and creative students. Mentoring these students, supporting them as they find their voices, and helping them grow into skilled researchers and communicators is extremely fulfilling. In short, intellectual curiosity brought me to science, but it’s the people (mentors, colleagues, students) that keep me here.

What scientific development are you currently most excited about?

Scientific advances that address the global challenges of climate change and renewable energy are at the top of my list. I’m particularly excited about the growing synergy among computational chemistry, experimental chemistry, and data science to harness the power of machine learning to discover and develop new molecules, materials, and processes for renewable fuels. In parallel with these and other exciting scientific advances in catalysis and photochemistry, I’m thrilled by the ever-growing presence of diversity, inclusion, and equity work in the scientific community. As the people doing science become increasingly diverse, the breadth and depth of scientific understanding and the advances that benefit society also grow.

What direction do you think your research field should go in?

As global population and the corresponding energy demand continues to surge, the urgent need for renewable energy requires creative solutions from the chemical community and concerted effort as citizen scientists. I believe the spectroscopy and photochemistry communities should be laser focused – pun intended – on environmental issues, especially renewable energy and climate change. I also believe that scientists should consider increased engagement in the public sector, helping to communicate science to a diverse array of audiences.

What attracted you to becoming an Editorial Board Member for *Communications Chemistry*?

One of the greatest things about being a chemist is the opportunity to engage with a vibrant scientific community where ideas are generated, exchanged, and evaluated. I was attracted to becoming an Editorial Board Member at *Communications Chemistry* because it had the potential to connect me with a broader chemical community and challenge me to keep learning and evolving as a scientist. For me, meeting the editorial team of talented, engaged, and collaborative professionals sealed the deal.

What have you gotten out of the experience of being an Editorial Board Member for *Communications Chemistry*?

In addition to being more connected to the broader chemical community and learning new areas of research beyond my sub-discipline, my work as an EBM has made me a better writer, evaluator, and teacher. For example, as an EBM I see how expressing the “so what” of a study (not just the “what”) is such an important and undervalued skill. I’ve translated this first-hand experience into more effective mentorship of my students in both the research and classroom settings.

What do you see as the role of *Communications Chemistry* in the scientific community?

*Communications Chemistry* is a venue for science without sub-disciplinary boundaries. If you have a significant idea, advance, or insight to share with the chemical community and you think colleagues in a variety of sub-disciplines would find it intriguing, then give CommsChem a try. From my perspective, *Communications Chemistry* also goes beyond publishing great science – it also supports the people engaged in the scientific endeavor though policies like open access publishing, transparent peer review, acknowledging reviewers’ work in reviewer of the month awards, and providing training grants for early career researchers. I also find the close collaboration between professional editors and Editorial Board Members to be exceedingly robust and beneficial for authors, as it encourages different perspectives on manuscript assessment and publishing decisions.

*This interview was conducted by the editors of Communications Chemistry.*

